# Pig castration: will the EU manage to ban pig castration by 2018?

**DOI:** 10.1186/s40813-016-0046-x

**Published:** 2016-12-20

**Authors:** Nancy De Briyne, Charlotte Berg, Thomas Blaha, Déborah Temple

**Affiliations:** 1 Federation of Veterinarians of Europe, Avenue Tervueren 12, 1040 Brussels, Belgium; 2 Department of Animal Environment and Health, Swedish University of Agricultural Sciences, POB 234, Skara, SE-532 23 Sweden; 3 German Veterinary Association for Animal Welfare, Wiesenweg 11, 49456 Bakum, Germany; 4 Universitat Autònoma de Barcelona, Veterinary School, Farm Animal Welfare Education Center, 08193 Bellaterra, Barcelona, Spain

**Keywords:** Piglet castration, Analgesia, Anaesthesia, Animal welfare, Immunocastration, Immunovaccination

## Abstract

**Background:**

In 2010, the ‘European Declaration on alternatives to surgical castration of pigs’ was agreed. The Declaration stipulates that from January 1, 2012, surgical castration of pigs shall only be performed with prolonged analgesia and/or anaesthesia and from 2018 surgical castration of pigs should be phased out altogether.

The Federation of Veterinarians of Europe together with the European Commission carried out an online survey via SurveyMonkey© to investigate the progress made in different European countries. This study provides descriptive information on the practice of piglet castration across 24 European countries. It gives also an overview on published literature regarding the practicability and effectiveness of the alternatives to surgical castration without anaesthesia/analgesia.

**Results:**

Forty usable survey responses from 24 countries were received. Besides Ireland, Portugal, Spain and United Kingdom, who have of history in producing entire males, 18 countries surgically castrate 80% or more of their male pig population. Overall, in 5% of the male pigs surgically castrated across the 24 European countries surveyed, castration is performed with anaesthesia and analgesia and 41% with analgesia (alone). Meloxicam, ketoprofen and flunixin were the most frequently used drugs for analgesia. Procaine was the most frequent local anaesthetic. The sedative azaperone was frequently mentioned even though it does not have analgesic properties. Half of the countries surveyed believed that the method of anaesthesia/analgesia applied is not practicable and effective. However, countries that have experience in using both anaesthesia and post-operative analgesics, such as Norway, Sweden, Switzerland and The Netherlands, found this method practical and effective. The estimated average percentage of immunocastrated pigs in the countries surveyed was 2.7% (median = 0.2%), where Belgium presented the highest estimated percentage of immunocastrated pigs (18%).

**Conclusion:**

The deadlines of January 1, 2012, and of 2018 are far from being met. The opinions on the animal-welfare-conformity and the practicability of the alternatives to surgical castration without analgesia/anaesthesia and the alternatives to surgical castration are widely dispersed. Although countries using analgesia/anaesthesia routinely found this method practical and effective, only few countries seem to aim at meeting the deadline to phase out surgical castration completely.

## Background

Many piglets in Europe are castrated surgically without any anaesthesia or post-operative analgesia. This is allowed by European legislation up to an age of 7 days [1]. Piglets are neurologically mature newborns such as lambs, kids, calves and human infants [2]. Such newborns mature animals usually become conscious within the first few minutes to hours after birth [3]. Castration is a painful and stressful procedure [4]. Some studies report behavioural alterations for several days after the procedure indicating that piglets likely experience postoperative pain [5–7], whereas results based on physiological measures have proven to be more inconsistent as reviewed by [8]. Although the use of anaesthetics [9, 10] would appear to be of benefit during the procedure itself, without the combined use of an analgesic, physiological responses to the procedure post-recovery would seem to indicate that the pain experienced is still great [4]. Castration of male pigs is hence a substantial animal welfare problem. To tackle this, in 2010, on the initiative of the European Commission and the Belgian Presidency, representatives of European farmers, meat industry, retailers, scientists, veterinarians – represented by the Federation of Veterinarians of Europe (FVE) and animal welfare Non-Governmental Organisations agreed upon the ‘European Declaration on alternatives to surgical castration of pigs’, from here on referred to as the Declaration [11].

The final goal of this Declaration is to phase out the surgical castration of pigs by 2018 in all European Union (EU) and all European Free Trade Association (EFTA) countries. But the Declaration also requested that from 1 January 2012, surgical castration of pigs shall only be performed with prolonged analgesia and/or anaesthesia.

In September 2015, FVE together with the European Commission decided to analyse the situation with respect to the progress seen in the different countries following up the Declaration. Specific focus was given to getting an overview of the situation regarding surgical castration with prolonged analgesia and/or anaesthesia in the different countries involved.

## Methods

This publication is based on an online survey, discussions with regional experts in pig castration and an investigation of (scientific) opinions on the different alternatives existing to surgical pig castration. The online survey on pig castration was designed by FVE and the European Commission, Directorate General for Health and Food Safety via SurveyMonkey©. It was distributed to all national veterinary organisations and to members of the European Association of Porcine Health Management (EAPHM) between 28 September 2015 and 30 October 2015. In total, 44 surveys from 24 countries were received and 40 of them provided usable answers. The final number of respondents per country varied from 1 to 5. Results were expressed at country level. Only consistent answers between respondents of the same country were considered. Each country was asked about the estimated percentage of i) castrated piglets; ii) castrated with analgesia and anaesthesia; iii) castrated with analgesia only; iv) castrated without analgesia or anaesthesia and v) immunocastrated piglets. The surveyed consisted of 3 open questions, 6 dichotomous and 3 multiple-choice questions (Table 1). After the survey, regional experts from 9 countries (pig veterinarians with publications or with known societal involvement in pig castration) were consulted to verify the survey answers and obtain more in-depth information on the situation in the different countries.

**Table 1:** Questions included in the survey on pig castration

*Open questions*
Percentage of pigs castrated
Percentage of pigs castrated with analgesia and anaesthesia
Percentage of pigs castrated with analgesia only
Percentage of pigs castrated without analgesia or anaesthesia
Percentage of immunocastrated pigs
List the anaesthetics and analgesics used for pigs in “your” country.
What are the main obstacles to reach the goals of the Brussels Declaration in “your” country?
*Dichotomous and multiple choice questions*
In the last 3–5 years, has the number of male piglets that are being castrated under anaesthesia and/or analgesia gone up in your country? (yes, no)
In the last 3–5 years, has the number of male piglets that are not castrated anymore gone up in your country? (yes, no)
In the last 3–5 years, has the number of male piglets that have been immunocastrated gone up in your country? (yes, no)
F1 - Who is allowed to administer anaesthesia/analgesia in your country? (only a vet, farmer)
F2 - Is the method of anaesthesia/analgesia applied practicable and effective? (yes, no)
F3 - In your country, how do you feel the government and stakeholders are working towards complying with the European declaration on pig castration (0: Little is done to meet the goals of the European declaration of pig castration; 1: Working towards it)
F4 - Has an official deadline to phase out castration been set in your country? (yes, no)
F5 - Economic impact of castration under the use of anaesthesia and/or prolonged analgesia and phasing out pig castration? (0: Neglectable / minor cost in relation to other costs; 1: Serious extra cost)
F6 - Welfare impact of castration under the use of anaesthesia and/or prolonged analgesia and phasing out pig castration? (0: negative; 1: neutral; 2: positive)

Three continuous variables were converted into dichotomous variables to look for possible associations between variables. Each country was classified in one of two categories based on expert opinion on the percentages provided by the survey:

i) surgically castrated piglets (0: 0–20%; 1: 80–100%), twenty three countries classified: Belgium was not considered here for having an intermediate percentage.
ii) castrated with analgesia and anaesthesia (0: 0–6%; 1: 24–99%), twenty four countries classified.
iii) castrated with analgesia only (0: 0–12%; 1: 72–99%). Twenty two countries classified: the Czech Republic and France were not considered here for having intermediate percentages of pigs castrated with analgesia only.

The ‘Genmod’ procedure for binomial data was applied to detect possible associations between the answers given to those three questions and the variables F1 to F6 present in the survey (Table 1). A *p*-value of 0.05 was considered significant for all analyses.

## Results

### Percentages of pigs castrated

**Fig. 1:**
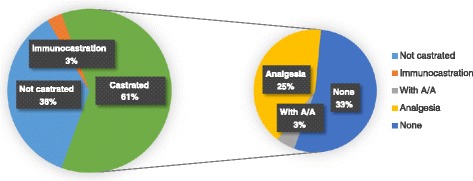
Percentage of male pigs castrated and methods of castration in the 24 surveyed countries

Table 2 shows the percentage of pigs castrated using different methods, according to the survey. In 18 out of the 24 countries that participated in the survey, 80% or more of male pigs are surgically castrated. In Ireland, Portugal, Spain, the Netherlands and United Kingdom, 20% or less of the male pigs are castrated. Looking at the size of the total pig population, this corresponds to 61% of male pigs being surgically castrated in Europe (Fig. 1). Belgium, France, Germany and Switzerland reported an increase in the number of entire raised males in the last 3–5 years and the Netherlands a strong increase.

**Table 2:** Percentages of entire males, immunocastrated and surgically castrated commercial piglets and methods of castration used in the 24 countries surveyed

Country (number of usable answers)	Entire males	Immuno castrated	Surgical Castration	Break-out surgical castration			Pig population*
				Castrated with analgesia & anaesthesia	Castrated with analgesia only (%)	Castrated without analgesia OR anaesthesia	
	% total	% total	% total	% total surgical	% total surgical	% total surgical	
Austria (2)	5	0	95	1	72	27	2869
Belgium (4)	15	18	67	3	6	91	6351
Czech (2)	5	5	90	6	31	63	1548
Denmark (4)	5	0	95	0	95	5	12402
Estonia (1)	0	0	100	0	10	90	359
Finland (1)	4	0	96	0.5	99	0.5	1258
France (4)	20	0	80	0	50	50	13428
Germany (1)	20	<1%	80	<1%	99	0	28046
Hungary (1)	1	0	99	0	0	100	2935
Iceland (1)	5	0	95	0	95	5	36
Italy (1)	2	5	93	0.5	2.5	97	8561
Ireland (1)	100	0	0	0	0	0	1468
Latvia (1)	0	0	100	0	0	100	368
Luxembourg (1)	1	0	99	0	99	1	90
Netherlands (1)	80	0	20	30	0	70	12013
Norway (1)	1	<1%	99	99	0	1	1644
Portugal (1)	85	2.5	12.5	0	0	100	2014
Romania (1)	0	5	95	2	4	94	5180
Slovakia (1)	0	10	90	0	12	88	637
Slovenia (1)	1	0	99	1	9	90	288
Spain (3)	80	5	15	1	7	92	25495
Sweden (2)	0	6	94	24	76	0	1478
Switzerland (2)	5	2.5	92.5	97	0	3	1573
UK (2)	98	<1%	2	4.5	4.5	91	4383
Europe-24 mean (median)		2.7 (0.2)	78 (95)	11 (0.5)	32 (7.5)	50 (65)	132920
Europe-24 (according to pig population)	36%	3%	61%	5% of the total of surgically castrated pigs	41% of the total of surgically castrated pigs	54% of the total of surgically castrated pigs	

^a^ In 1000 heads- data from Eurostat 2013 except Norwegian pig population data from NorwegianNational Bureau of Statistics 2015

Norway, Switzerland, The Netherlands and Sweden reported 99, 97, 30 and 24% of surgically castrated animals with both anaesthesia and analgesia, respectively. In the other countries and according to the survey, less than 6% of the piglets were castrated using anaesthesia and analgesia.

According to the survey, seven countries castrate surgically more than 70% of the male piglets in their country using analgesia (alone). In France and Czech Republic, 50 and 31% of piglets respectively were castrated surgically using analgesia. The other countries reported the administration of analgesia in 10% or less of male pigs castrated. In the last 3–5 years, Austria, Denmark, Finland, France, Germany, Iceland and Luxemburg noticed an increase in the number of piglets castrated under anaesthesia and/or analgesia.

The mean percentage of immunocastrated pigs in the countries surveyed was 2.7% (median 0.2%; range = 0–18%) with Belgium having the highest estimated percentage of immunocastrated pigs. Respondents from Belgium, Czech Republic, Norway, Romania, Spain and Sweden reported an increased number of immunocastrated pigs during the last 3–5 years.

### Products used for analgesia and anaesthesia in pigs

#### Using analgesia/anaesthesia: how practical and effective are they?

Respondents were asked whether the method of anaesthesia/analgesia applied is practicable and effective. Overall, in 50% of the countries respondents answered “no” and in 37% they answered “yes”. 9% of the countries did not have a consistent answer between the respondents and 4% did not answer.

Nine experts also commented via free text that they felt that the use of analgesia alone (often not prior, but only at the time of the surgery) is insufficient for avoiding stress and pain for the piglets. According to the survey only veterinarians are allowed to administer anaesthesia/analgesia in 67% of the countries. In the Netherlands, Sweden and Switzerland these medicines are used under veterinary prescription but the farmer is allowed to administer them. In some countries (e.g. Sweden), farmers first have to pass a specific training course. In Denmark and France, veterinarians can prescribe analgesia for farmers, who are allowed to administer it, but anaesthetics must be administered by a veterinarian.

### Pig castration: how much is it of importance in the different countries?

In respect to how hard the government and stakeholders are working towards complying with the Declaration on pig castration, in 62% of the countries respondents replied that they felt that the government and stakeholders are working towards complying with the Declaration.

Regarding the question about whether in their country an official deadline on pig castration had been set, no countries, except of Germany and Norway, noted that a national deadline had been set. Respondents from the Czech Republic, the United Kingdom and Switzerland gave different answers. In some countries, experts noted that while the government had set no official date, some farm assurance systems had set deadlines. Several countries also noted that while no date had been set to phase out pig castration, they had a date set demanding analgesia (e.g. Finland has had an industry requirement since 2011, Denmark an industry requirement since 2009 and a legal requirement since 2011) or demanding the use of analgesia and anaesthesia (e.g. Sweden from 1 January 2016).

Regarding the economic impact of castration under the use of anaesthesia and/or analgesia and phasing out pig castration, in 38% of the countries respondents believe that the use of anaesthesia and analgesia causes considerable extra costs.

Regarding the welfare impact of castration under the use of anaesthesia and/or analgesia and phasing out pig castration, respondents from 67% of the countries surveyed were positive or very positive about the animal welfare benefits. One country thought that the welfare impact would be negative. The remaining respondents were either neutral or gave inconsistent answers.

According to the respondents and independently of their country, the main obstacles to reach the goals of the Declaration were the economic implications (mentioned 19 times were that extra costs occur to the farmer which are not be paid back by the consumer), the extra work load caused by using anaesthesia/analgesia (mentioned 11 times), the lack of practical and effective anaesthesia/analgesia protocols (mentioned 10 times), the lack of EU acceptance of entire males both by the market as by slaughterhouses (mentioned 7 times), risk of boar taint in meat (mentioned 3 times) and welfare problems associated with raising entire males (mentioned 2 times).

### Associations between variables from the survey

The use of analgesia and anaesthesia in pig castration was significantly associated with whether or not the producer is allowed to administer anaesthesia (*P* = 0.02). From the four countries that uses analgesia and anaesthesia in more than 20% of the pigs castrated, The Netherlands, Sweden and Switzerland allow the producer to administer analgesia and anaesthesia. Norway was the only country where analgesia and anaesthesia was frequently used to castrate piglets (99% castrated with analgesia and anaesthesia) but where the producer could not administer such products. The four countries that have experience in using analgesia and anaesthesia found this method practical and effective (*P* = 0.005).

The use of analgesia was associated with whether or not the country is working towards complying with the EU legislation (*p* = 0.03). The countries where respondents agreed that “little is done to meet the goals of the Declaration” did not use analgesic to castrate the majority of the piglets.

## Discussion of the survey results

For most countries, reliable statistical data on the amount of pigs castrated and on the methods used to castrate them is not available. The present survey by the FVE relied upon the answers of experts in pig production from different countries. Therefore, while the results presented in this document indicate the situation of each country in terms of pig castration, it should be recognised that this might not reflect the situation in the whole of Europe, nor give a complete picture.

Ireland, United Kingdom, Spain and Portugal have a history in producing entire males. In the Netherlands, now also the great majority of pigs produced are entire males (80%). The remaining 19 countries that participated to the survey castrated more than 80% of their male pig population. The ultimate goal of the Declaration [11], namely to phase out surgical pig castration, is therefore far from being reached by 2018.

Several countries agreed on deadlines with respect to banning surgical castration without analgesia and/or anaesthesia (Table 3). No country, however, has set a deadline to completely phase out surgical castration.

**Table 3:** Overview of deadlines in a selected number of countries

Country	Year	Deadline content
Denmark	2009, 2011	Ban on surgical pig castration without analgesia, industry requirement since 2009, legal requirement since 2011
Germany	2019	Ban on surgical pig castration without anaesthesia
Netherlands	2009	Ban on surgical pig castration without anaesthesia
Norway	2002	Ban on surgical pig castration without analgesia and anaesthesia
Sweden	2016	Ban on surgical pig castration without analgesia and anaesthesia
Switzerland	2010	Ban on surgical pig castration without anaesthesia

On average 5% of the male pig population surgically castrated across the 24 European countries surveyed was castrated with anaesthesia and analgesia; 41% with analgesia (alone) and 54% was castrated completely without any anaesthesia or analgesia. In 2010, it was estimated that 79% of the piglets were castrated without anaesthesia or analgesia [12].

Based on these results, there is still a major bottleneck in the use of the combination of anaesthesia and analgesia, the anaesthesia being the biggest constraint. Results from the PIGCAS project published in 2009 [13] indicated as well that in most countries, anaesthesia was not used and that analgesia was used even more seldom than anaesthesia. The use of analgesics (alone) for male pig castration has hence increased in the last few years.

### Expert opinion on surgical castration with analgesia and anaesthesia

The number of authorised and licensed analgesics and anaesthetics for pig castration is limited and differs largely between countries as can be seen in Table 4. For surgical castration of male piglets to be used at farm level, the method must be easy to run without requiring expensive equipment while resulting in a significant reduction of pain for the piglets [14].

**Table 4:** Overview of the products used for analgesia and/or anaesthesia in pigs in the different countries according to the answers collected in the survey

Country	Trade name	Active substance	Marketing authorization holder
Austria	Finadyne	flunixin meglumine	MSD Animal Health
	Melovem	meloxicam	Dopharma
	Metacam	meloxicam	Boehringer Ingelheim
	Narketan	ketamine	Vétoquinol AG
	Stresnil	azaperone	Provet AG
Belgium	Metacam	meloxicam	Boehringer Ingelheim
	Ketamidor	ketamine	Richter farma
	Stresnil	azaperone	Eli Lilli
	Novocain	procaine hydrochloride, Procaine + adrenaline	Kela laboratoria/VMD
Czech Republic	Narketan	ketamine	Vetoquinol, Bioveta
	Stresnil	azaperone	Eli Lilli
	Procamidor	procaine	Richter Pharma AG
Denmark	Finadyne	flunexin	MSD Animal Health
	Melovem	meloxicam	Sacnvet
	Ketador	ketamine	Salfarm
	Procamidor	procaine hydrochloride	Salfarm
	Coxofen	ketoprofen	Dechra
	Romefen	ketoprofen	Merial
	Rifen	Ketoprofen	Salfarm
	Metacam	meloxicam	Boehringer Ingelheim
	Meloxidolor	meloxicam	Huvepharma
Estonia	Porcamidor	procaine	Richter pharma
France	Stresnil	azaperone	Eli Lilly
	Metacam, Melovem	meloxicam	Boehringer Ingelheim
	Imalgene	ketamine	Merial
	Finadyne	Flunixine	MSD Animal Health
	Procamidor	procaine hydrochloride	Richter farma
Germany	Ursotamin	ketamine	Medistar Arzneimittelvertrieb
	Stresnil	azaperone	Elanco animal Health
	Metacam	meloxicam	Boehringer Ingelheim, Vetmedica
Hungary	Finadyne	flunixin	Intervet
	Melovem	meloxicam	Dopharma International
	Minocain	procaine	Kon-Pharma
	Ketofen, Ketolodor, Ketanest, Ketamidor, Ketink	ketoprofenum	Merial, Le Vet Beheer B.V., Bela-pharm GmbH and Co.KG, Richter Pharma, Industrial Veterinaria S.a.
	Stresnil	azaperone	Eli Lilly Benelux N.V.
Iceland	Metacam	meloxicam	Boehringer Ingelheim
	Procamidor	procaine hydrochloride	Boehringer Ingelheim
Ireland	Metacam	meloxicam	
	Tolfine	tolfenamic	Vetoquinol
	Anaestamine Ketamidor	ketamine	Le Vet Beheer B.V Richter Pharma
	Stresnil	azaperone	Elanco
	Flunazine	flunixine	Cross Vetpharm Group Limited
Italy	Metacam	meloxicam	Boehringer Ingelheim
	Tolfedine	tolfenamic acid	Vetoquinol
	Stresnil	azaperone	Elanco animal Health
		lidocaine	
	Alivios	flunixin meglumine	Fatro
Latvia	Ketofen Ketodolor Dinalgen Ketink Rifen	Ketoprofenum	Merial Le Vet Laboratorios Dr. Esteve Industrial Veterinaria. - Richter Pharma
	Aniketam Ketamidor	ketamine	Le Vet Beheer B.V Richter Pharma AG
	Alfacilline Procamidor	procaine hydrochloride	Alfasan International Richter Pharma
	Sodium Salicyl	sodium salicylate	Dopharma Research
	Novasul	metamizole	Richter Pharma
	Pracetam	paracetamol	Ceva Sante Animale
Luxembourg	same as Belgium		
Netherlands	Novem	meloxicam	Boehringer Ingelheim
	Castralgin	metamizole	Interchemie de Adelaar
	Gas	CO2 O2	
	Anaestamine Ketamidor Narketan	ketamine	Le Vet Beheer B.V Richter Pharma Vetoquinol
	Procamidor Pronestesic	procaine hydrochloride	Richter Pharma Fatro S.P.A.
Norway	Lidokain 20 mg/ml- adrenalin 5	lidocaine	NAF Apotek
	Lidokel-Adrenalin vet		Kela
	Procamidor	procaine hydrochloride	Richter pharma
	Metacam	meloxicam	Boehringer Ingelheim
	Loxicom	meloxicam	Norbrook
Romania	Stresnil	azaperone	Janssen Pharmaceutica
Slovakia	Stresnil	azaperone	Janssen Pharmaceutica
Slovenia	Bioketan	ketamine	Vetconsult
	Novasul	metamizole	Vetconsult
Spain	Ketolar	ketamine	Parke-Davis
	Zoletil	tiletamine + zolazepam	Virbac
	Stresnil	azaperone	Esteve
	Valium	diazepam	Roche
	Metacam	meloxicam	Mylan Pharmaceuticals
	Meloxidyl	meloxicam	Ceva
	Procamidor	procaine hydrochloride	Richter pharma
	Ketoprofeno	ketoprofen	
Sweden	Melovem	meloxicam	Salfarm Scandinavia
	Metacam	meloxicam	Boehringen Ingelheim
	Xylocain	Lidocaine	AstraZeneca
Switzerland	Metacam	meloxicam	Boehringer Ingelheim
	Stresnil	azaperone	Ketavet, Janssens
	Janssen	ketamine	Graeub
	Dolorex	butorphanol	Intervet
	Narketan	ketamine	Vétoquinol
	Isoflurane	isoflurane	
UK	Ketamidor	ketamine	Richter Le Vet Beheer B.V.
	Stresnil	azaperone	Eli Lilly
	Solacyl	sodium salicylate	Dechra, Eurovet
	Finadyne, Allevinix, Pyroflam, Flunixin	flunixine	Intervet, Merial, Norbrook
	Kefotem	ketamine	
	Meloxidyl Metacam, Novem Inflacam, Rheumacam Recocam Melovem Emdocam Meloxidolor Loxicom Contacera	meloxicam	Ceva Boehringer Ingelheim Chanelle Cross VetPharm Dopharma Emdoka Le Vet Norbrook Zoetis

Some products may be missing and some products are used off-label

Procaine was the most cited local anaesthetic among the countries surveyed. Even though lidocaine is by far the most common local anaesthetic tested in experimental studies [8], this local anaesthetic was only mentioned in Italy, Norway and Sweden. Local administration of lidocaine has been shown to reduce the cortisol level measured 20 min after castration and has shown to reduce the movements and intensity of vocalisation during castration [15]. The anaesthetic effectiveness of lidocaine under experimental conditions has been reviewed [8] and found not to be immediate and limited in duration. In case of the use of intratesticular injection of lidocaine with adrenaline, it takes the lidocaine 3 min to reach the testicular cordons [16]. Lidocaine does not readily diffuse through the tunica vaginalis and in the cremaster muscle which can explain the nociceptive response to surgical castration under local anaesthesia [17]. In our survey, meloxicam, ketoprofen and flunixin were the most frequently cited analgesics across countries. Those three drugs are non-steroidal anti-inflammatory drugs (NSAIDs). Their effectiveness in alleviating pain during male pig castration is questionable. Some studies show that pre-emptive administration of meloxicam, 30 min before the procedure, gives some post-operative analgesia after surgical castration [18]. However, others [10] reported very limited effects of meloxicam in reducing pain related to pig castration. SUIVET [19], an organisation of pig veterinarians in Italy, proposed a protocol for pig castration using a combination of meloxicam and procaine. To give the product time to become efficient, they suggest giving the injection first to 5 litters, after which to come back to castrate the piglets. In order to limit the number of injections, they suggest to combine it with the iron injection usually provided anyway [19]. Ketoprofen did not show any effect on pain responses during castration, but postoperative pain was reduced in these piglets in terms of scratching, tail wagging and isolating themselves on the day after castration [20].

General anaesthesia can be induced by use of inhalation agents or injection. The use of inhalation agents was mentioned by the Netherlands (Carbone dioxide) and Switzerland (Isoflurane). The availability of injection anaesthetics for general anaesthesia was mentioned in several countries. General anaesthesia has advantages but is difficult to practice at the farm level and present some major drawbacks [15, 21]. The use of CO_2_ is very controversial. Piglets castrated under CO2 anaesthesia display more interactive behaviours during the 8 day observation period, however the piglets that were castrated under anaesthesia also displayed behaviours indicative of pain and discomfort up 6 days after castration [22].

Although CO_2_ is very commonly used for pre-slaughter stunning, due to a lack of alternatives, C0_2_ produces strong aversion (irritation and asphyxia) in pigs before they lose consciousness [23, 24]. Isoflurane inhalation was found in one large scale study to only have given sufficiently anaesthesia in 77% of the piglets [25].

The sedative azaperone was frequently mentioned. Sedation makes the piglets easier to handle, however it is not effective at all in relieving pain. It may be used as premedication to local and general anaesthesia such as in combined use with ketamine [26].

Half of the countries surveyed believe that the method of anaesthesia/analgesia applied is not practicable and effective. Extra cost, extra work load and the lack of practical and effective protocols were 3 main constraints identified by the respondents. One study [20] estimated that local anaesthesia prior to castration increase the labour demand by 39 to 52%. Still, countries that have some experience in using analgesia and anaesthesia (Norway, Switzerland, The Netherland and Sweden) found their method practical and effective. Furthermore, based on the survey, that a producer is allowed to administer anaesthesia and analgesia seems to facilitate the use of such products in a routine basis to castrate piglets. In the Netherlands, Sweden and Switzerland the farmer is allowed to administer anaesthesia and analgesia. In Norway, farmers cannot use analgesia and anaesthesia. In some other countries as Denmark and France, veterinarians can prescribe analgesia to be administered by farmers, but anaesthetics must be administered by a veterinarian. In Sweden, a farmer may inject local anaesthesia and analgesia to perform pig castration, when he has attended both a course in handling pharmaceuticals and a course in correct administration of scrotal local anaesthesia. According to FVE’s Veterinary Act [27] and most national Veterinary Acts, administering anaesthesia and doing surgery entering a body cavity is a task that can only be performed by veterinarians. Potential complications associated with surgical castration include haemorrhage, excessive swelling or oedema, infection, poor wound healing, and failure to remove both testicles and risks involved with anaesthesia when used [28]. Therefore, FVE, the Federation of veterinarian of Europe, is of the position that pig castration should always be performed by a veterinarian under general or local anaesthesia with additional prolonged analgesia [29]. It should also be noted that some anaesthetics such as ketamine in many countries is upon strict regulation due to illicit use.

As a priority to make further progress, a series of mutually agreed, practical and effective analgesia and/or anaesthesia protocols should be agreed at a national or EU level. These protocols should be cost effective, produce minimum stress and pain both during and after castration and be safe for both the handler and the piglet. The method should also ensure a quick recovery to minimize the risk of the piglet being crushed by the sow.

In 2016, a European consortium on the basis of a call of the European Commission (SANCO/2014/G3/026) started a study on methods of pig castration – called ‘CASTRUM’. More specifically the study will try to identify available methods for the use of anaesthesia and/or prolonged analgesia and specifically look into alternatives to surgical castration for ‘heavy’ pigs used in traditional products. The outcome of this study should become available in 2017.

### Expert opinion on immunocastration

The estimated average percentage of immunocastrated pigs in the countries surveyed was 2.7% (median = 0.2%), where Belgium presented the highest estimated percentage of immunocastrated pigs (18%). Respondents from Czech Republic, Norway, Romania, Spain and Sweden reported a slight increase in immunocastrated pigs in the last 3–5 years. Immunocastration has been permitted in the EU since 2009, but while it is used to a great extend in some countries abroad such as Australia [30], it seems still difficult to break through in Europe. Immunocastration is used in a higher proportion of pigs in Belgium mainly due to the impact of a major Belgian retailer (Colruyt) who since end 2010 only accepts pork from pigs castrated by vaccination. At this moment Zoetis is the only company which has a Gonadotrofine Releasing Factor vaccine on the market (Improvac R). In terms of feasibility, the vaccine requires two doses, at least 4 weeks apart, with the second dose being given ideally 3–4 weeks before slaughter. Pigs slaughtered at a higher slaughter weight may need more than two doses. A single effective shot of the “vaccine” is being investigated at the moment [31]. Immunocastration eliminates the acute pain experienced by surgically castrated piglets; however welfare concerns still arise due to the fact that immuno-castrated pigs behave as entire males until the second vaccination. The main limitation to immunocastration is linked to market issues and human error (vaccinating outside the recommended time period, missing a dose [32]. Most retailers do not accept pork from immunocastrated pigs being afraid for poor public acceptance. However, in the case of Belgium, the acceptance of immunocastration led to a better welfare-friendly image of the retailer and large scale surveys conducted in European countries, showed that over 60% of surveyed consumers informed about the issue preferred immunocastration to surgical castration with anaesthesia [33].

From an animal-ethical point of view, not all alternatives to pig castration are equal [34, 35]. Immunocastration may give the greatest benefit to the animals, while raising entire males can still lead to pigs suffering from aggressive behaviour amongst each other and giving pain relief are seen as less animal-friendly alternative [34].

### Expert opinion on entire males

Entire males’ production is another main alternative to surgical castration. From an animal welfare perspective, raising entire males has benefits but also disadvantages [30]. In Ireland, Portugal, Spain and the United Kingdom less than 20% of the pigs were surgically castrated. Most countries do not rear entire male pig due to the incidence of boar taint. There is so far no international accepted and validated on-line method available for the measurements of boar taint in carcasses that throughout fulfils the requirement for a highly streamlined industry at the slaughterhouses [36]. Ireland and the United Kingdom address the incidence of boar taint by slaughtering at low weight and before sexual maturity. According to de Roest [37], the raising of entire males can be an interesting option for many countries, except for countries and production systems with a high age at slaughtering. The costs and benefits of this alternative will depend on the percentage of males with boar taint at slaughtering. Raising entire males should not generate more than 2.5% of boar taint among slaughter pigs, in order to maintain the considerable economic benefits of better feed efficiency of entire males with respect to castrate [37].

## Conclusions

The deadline of 1 January 2012, which marks the day after which all castrated piglets reared in the EU and EFTA countries have to be treated with prolonged analgesia and/or anaesthesia, is far from being met in the majority of the 24 countries we surveyed. Analgesia alone is now used in several countries, probably partly due to the Declaration, but the effectiveness of this method to alleviate the pain during male piglet castration is questionable. There is still a major bottleneck in the use of the combination of anaesthesia and analgesia among the majority of the countries surveyed, the anaesthesia appearing to be the biggest constraint at the farm level.

The percentage of male pig population immunocastrated is still very low. Still, it appears as a promising alternative to surgical castration in countries such as Belgium. In Ireland, United Kingdom, Spain and Portugal, the production of entire males has been for long used as the main type of pig meat and a further increase is foreseen in other countries. In our survey Belgium, France, Germany, the Netherlands and Switzerland reported an increase in the number of pigs raised as entire males. Depending of the country, immunocastration and entire male production are foreseen as valuable alternatives to surgical castration.

As a priority to make further progress, a series of practical and effective analgesia and/or anaesthesia protocols should be mutually agreed at a national or EU level.

It is the apprehension of the authors that given the current economic climate, it is unlikely that pig producers will be able to follow the Declaration on pig castration unless it becomes mandatory in one way or another.

## Abbreviations

Declaration: European Declaration on alternatives to surgical castration of pigs
EAPHM: European Association of Porcine Health Management
EU: European Union
FVE: Federation of Veterinarians of Europe.
